# Transcriptomic and Metabolomic Analyses Reveal a Potential Mechanism to Improve Soybean Resistance to Anthracnose

**DOI:** 10.3389/fpls.2022.850829

**Published:** 2022-04-27

**Authors:** Longming Zhu, Qinghua Yang, Xiaomin Yu, Xujun Fu, Hangxia Jin, Fengjie Yuan

**Affiliations:** Zhejiang Key Laboratory of Digital Dry Land Crops, Institute of Crop and Nuclear Technology Utilization, Zhejiang Academy of Agricultural Sciences, Hangzhou, China

**Keywords:** soybean, anthracnose, *Colletotrichum truncatum*, transcriptomics, metabolomics, resistance mechanism

## Abstract

Anthracnose, caused by *Colletotrichum truncatum*, leads to large-scale reduction in quality and yield in soybean production. Limited information is available regarding the molecular mechanisms of resistance to anthracnose in soybean. We conducted a transcriptomic and targeted metabolomic analysis of pods from two soybean lines, “Zhechun No. 3” (ZC3) and ZC-2, in response to *C. truncatum* infection. Factors contributing to the enhanced resistance of ZC-2 to anthracnose compared with that of ZC3, included signal transduction (jasmonic acid, auxin, mitogen-activated protein kinase, and Ca^2+^ signaling), transcription factors (*WRKY* and *bHLH*), resistance genes (*PTI1*, *RPP13*, *RGA2*, *RPS6*, and *ULP2B*), pathogenesis-related genes (*chitinase* and *lipid transfer protein*), and terpenoid metabolism. Targeted metabolomic analysis revealed that terpenoid metabolism responded more promptly and more intensely to *C. truncatum* infection in ZC-2 than in ZC3. *In vitro* antifungal activity and resistance induction test confirmed that jasmonic acid, auxin signaling and terpenoids played important roles in soybean resistance to anthracnose. This research is the first study to explore the molecular mechanisms of soybean resistance to anthracnose. The findings are important for in-depth analysis of molecular resistance mechanisms, discovery of resistance genes, and to expedite the breeding of anthracnose-resistant soybean cultivars.

## Introduction

Anthracnose, caused by *Colletotrichum truncatum* (Schwein.) Andrus and W.D. Moore, is a destructive fungal disease of soybean [*Glycine max* (L.) Merr.] in subtropical and tropical regions (Hyde et al., 2009). Aboveground organs of soybean plants, including the cotyledons, stems, pods, petioles, veins, and leaves can be infected at any stage of development by *C. truncatum* (Hartman et al., 2015). Infection severely affects soybean growth and development (Rogério et al., 2017; Tian et al., 2017; Dias et al., 2019). Anthracnose infection is favored by high temperature and moisture. Hence, this disease causes significant losses in soybean crops in subtropical and tropical regions, including the southern soybean-growing areas of China and the United States, as well as northern Argentina, Brazil, Thailand, and India (Wrather et al., 2010; Mahmodi et al., 2013; Dias et al., 2016, 2019; Nataraj et al., 2020).

To prevent disease development and limit pathogen spread during soybean production, rapid diagnosis, biological control, sowing pathogen-free seeds, and chemical control are currently employed (Shovan et al., 2008; Tian et al., 2017). Compared with these control methods, the cultivation of disease-resistant cultivars is more economic, effective, and environmentally friendly. Thus, the breeding of anthracnose-resistant cultivars is of particular importance. To date, the research focus has predominantly been on screening soybean germplasm for anthracnose resistance. Thus, the anthracnose resistance of modern soybean cultivars from the United States, China, Brazil, and India has been evaluated (da Costa et al., 2009; Yang and Hartman, 2015; Nataraj et al., 2020; Feng et al., 2021). These studies detected no immune lines, and only a small number of resistant lines have been identified. Two major genes that interact in a complementary fashion are reported to contribute to anthracnose resistance (Nataraj et al., 2020), although the resistance genes have not yet been identified. Identification of resistance genes and an improved understanding of resistance mechanisms will help to accelerate the breeding of resistant cultivars.

“Multi-omics” approaches are powerful tools for resistance gene discovery and analysis of molecular defense mechanisms for soybean diseases other than anthracnose. For example, an integrated transcriptomic and metabolomic analysis revealed the regulation of soybean primary metabolism in response to Rhizoctonia foliar blight disease (Copley et al., 2017). Several important secondary metabolites and the expression profile of the regulatory genes that contribute to soybean resistance to *Phytophthora sojae* were identified by transcriptomic and metabolomic analysis (Zhu et al., 2018). A unique type of isoflavone *O*-methyltransferase, GmIOMT1, which participates in the induction of glycitein biosynthesis in soybean in response to infection by *Aspergillus oryzae* and *Rhizopus oligosporus*, was identified based on an integrated transcriptomic and metabolomic analysis (Uchida et al., 2020).

In this study, we conducted a transcriptomic and targeted metabolomic analysis of two soybean lines, “Zhechun No. 3” (ZC3) and ZC-2, the latter derived from irradiation of ZC3, in response to *C. truncatum* infection using RNA sequencing (RNA-Seq) and ultra-high-performance liquid chromatography–mass spectrometry (UHPLC-MS). Use of these approaches provided molecular insight into the transcriptional- and metabolic-level mechanisms of soybean resistance to anthracnose, and the possible factors that contribute to the enhanced resistance of ZC-2 to anthracnose compared with that of its wild-type parent ZC3. The results provide a foundation for elucidation of the resistance mechanisms and the breeding of anthracnose-resistant soybean cultivars.

## Materials and Methods

### Plant Materials

The soybean (*Glycine max* L.) lines “Zhechun No. 3” (ZC3) and ZC-2 were used in this study. ZC-2 is a mutant that was identified among progeny derived from ZC3 seeds irradiated with 150 Gy gamma rays (Yuan et al., 2007). Seeds of each soybean line were sown in sterilized nutrient soil in plastic pots (35 cm diameter) and transferred to a pre-sterilized greenhouse. Three seedlings were retained in each pot. Twenty pots were prepared for each soybean line.

### *Colletotrichum truncatum* Culture and Inoculum Preparation

The *C. truncatum* isolate CT5 (Feng et al., 2021) was cultured on potato dextrose agar (PDA) at 25°C in the dark for 5 days. The mycelial suspension used as inoculum was prepared as follows. Ten mycelial disks (5 mm diameter) from an actively growing culture of isolate CT5 on PDA were added to potato dextrose broth (PDB) in 500 mL flasks and cultured for 4 days in an incubator at 25°C with shaking at 120 rpm in the dark. After incubation, the PDB was filtered through sterilized gauze. The mycelial pellets were rinsed with sterilized water at least six times to remove as much residual PDB as possible. The residual water was squeezed out and the pellets were weighed. The pellets were resuspended in sterilized water, fragmented in a blender at low speed for 20 s, and diluted with sterilized water to a concentration of 50 mg mL^–1^. All steps were conducted on an ultra-clean workbench.

### Sample Preparation

Healthy pods of uniform maturity (approximately 15 days after flowering) from each soybean line were sampled, quickly transported to the laboratory, and washed with sterile water. The pods were placed in a beaker containing 500 mL mycelial suspension, gently stirred for 10 s, and then removed from the suspension. After draining off the excess mycelial suspension, the inoculated pods were placed on filter paper in 15 cm petri dishes containing 3 mL sterilized water. As a control, pods were mock-inoculated using sterile water. The petri dishes were placed in an incubator at 25°C in the dark. Samples of inoculated and corresponding control pods for transcriptomic and metabolomic analysis were collected at 4, 8, 12, 24, and 48 h post-inoculation (HPI), immediately frozen in liquid nitrogen, and stored at −80°C. Three replicates for RNA-Seq and six replicates for UHPLC-MS were prepared. Each replicate comprised at least 15 pods. Symptoms of anthracnose on pods of soybean lines ZC-2 and ZC3 at 2 and 5 days after inoculation with *Colletotrichum truncatum* were recorded. Disease blotches were scanned, and their areas were calculated using the LA-S plant analysis system (Wseen, Shenzhen, China).

### Transcriptome Sequencing and Data Analysis

Total RNA was extracted using TRIzol Reagent (Invitrogen, Carlsbad, CA, United States) in accordance with the manufacturer’s protocol. The RNA quality was assessed using an Agilent 2100 Bioanalyzer (Agilent Technologies, Palo Alto, CA, United States) and RNase-free agarose gel electrophoresis. RNA library preparation and sequencing were performed by the Gene *Denovo* Biotechnology Co. (Guangzhou, China) on an Illumina HiSeq 2500 platform.

Raw reads were filtered with fastp v0.18.0 to obtain high-quality clean data. An index of the soybean reference genome (Wm82.a2) was constructed and paired-end clean reads were mapped using HISAT2.2.4 (Kim et al., 2015) with the default parameters. The fragments per kilobase of transcript per million mapped reads (FPKM) value was calculated to quantify the expression abundance and variation of genes using StringTie v1.3.1 (Pertea et al., 2015, 2016). Differential expression analysis was performed using DESeq2 and edgeR (Robinson et al., 2010; Love et al., 2014). Genes with false discovery rate < 0.05 and log_2_ expression fold change ≥1 were considered to be differentially expressed genes (DEGs). Principal component analysis (PCA) was performed with the gmodels R package^1^ to evaluate the relationships among samples and replicates. A statistical Kyoto Encyclopedia of Genes and Genomes (KEGG) pathway enrichment analysis for the DEGs was performed using KOBAS software (Kanehisa et al., 2008).

### Quantitative Real Time-PCR

To determine the first sampling time point, the expression of nine disease-responsive genes was detected by qRT-PCR. In addition, 24 DEGs were selected for qRT-PCR analysis to validate the RNA-Seq data. The methods for qRT-PCR analysis and statistical tests were performed as described previously (Jin et al., 2021). The specific primers used are listed in Supplementary Table 1.

### Ultra-High-Performance Liquid Chromatography–Mass Spectrometry and Data Analysis

Determination of the relative content of terpenoids was performed using UHPLC-MS. Extraction and sample preparation were performed in accordance with a protocol described previously (Chen et al., 2013; Doppler et al., 2016). The UHPLC separation was performed on an ExionLC System (Sciex Technologies, Redwood City, CA, United States). The mobile phase A was 0.1% formic acid in water and the mobile phase B was acetonitrile. The column temperature was set at 40°C. The auto-sampler temperature was set at 4°C and the injection volume was 2 μL. A Sciex QTrap 6500 + LC-MS/MS system (Sciex Technologies, Redwood City, CA, United States) was used for assay development. Typical ion-source parameters were as follows: IonSpray voltage, +5500/−4500 V; curtain gas, 35 psi; temperature, 400°C; ion source gas 1, 60 psi; ion source gas 2, 60 psi; and declustering potential, ±100 V.

SCIEX Analyst Work Station v1.6.3 software (Sciex Technologies, Redwood City, CA, United States) was employed for multiple-reaction monitoring for data acquisition and processing. The MS raw data (.wiff) files were converted to TXT format using MSconventer. An in-house R program and database were used for peak detection and annotation. The SIMCA 16.0.2 software package (Sartorius Stedim Data Analytics AB, Umeå, Sweden) was used for orthogonal projections to latent structures–discriminate analysis (OPLS-DA). The variable importance in the projection (VIP) value of the first principal component in the OPLS-DA analysis was determined. The metabolites with VIP > 1 and *p* < 0.05 (Student’s *t*-test) were considered to be significantly differentially accumulated metabolites (DAMs). The integrated analysis of transcriptomic and metabolomic data was conducted on the basis of the enriched KEGG pathways.

### *In vitro* Antifungal Activity and Resistance Induction Test for Terpenoids or Phytohormones

Eight terpenoids or phytohormones (simvastatin, 11-keto-beta-boswellic acid, cantharidin, picrocrocin, 3-indolebutyric acid, abscisic acid, methyl jasmonate, and *trans*-zeatin riboside) were randomly selected and tested for their antifungal activity and ability to induce anthracnose resistance. PDA medium was supplemented individually with these standard biochemical reagents (final concentration 10 μM); standard PDA medium was used as the control. Each plate was inoculated with a *C. truncatum* mycelium plug (5 mm in diameter). Each treatment comprised 20 plates. After culture at 25°C in the dark for 6 days, the plates were scanned and the colony area was calculated with the LA-S analysis system (Wseen, Shenzhen, China).

Healthy pods of ZC3 of uniform maturity were sampled. The pods were sprayed with an aqueous solution (10 μM) of one of the standard reagents, sterile water was used as the control. After moisturizing at 25°C for 1 h, the pods were rinsed with sterile water and inoculated with *C. truncatum*, as described above. Fifty pods were treated for each treatment. After incubation for 5 days, the disease blotches on the pods were scanned and the disease area was calculated with the LA-S analysis system (Wseen, Shenzhen, China).

## Results

### ZC-2 Is More Resistant to Anthracnose Than ZC3

**FIGURE 1:**
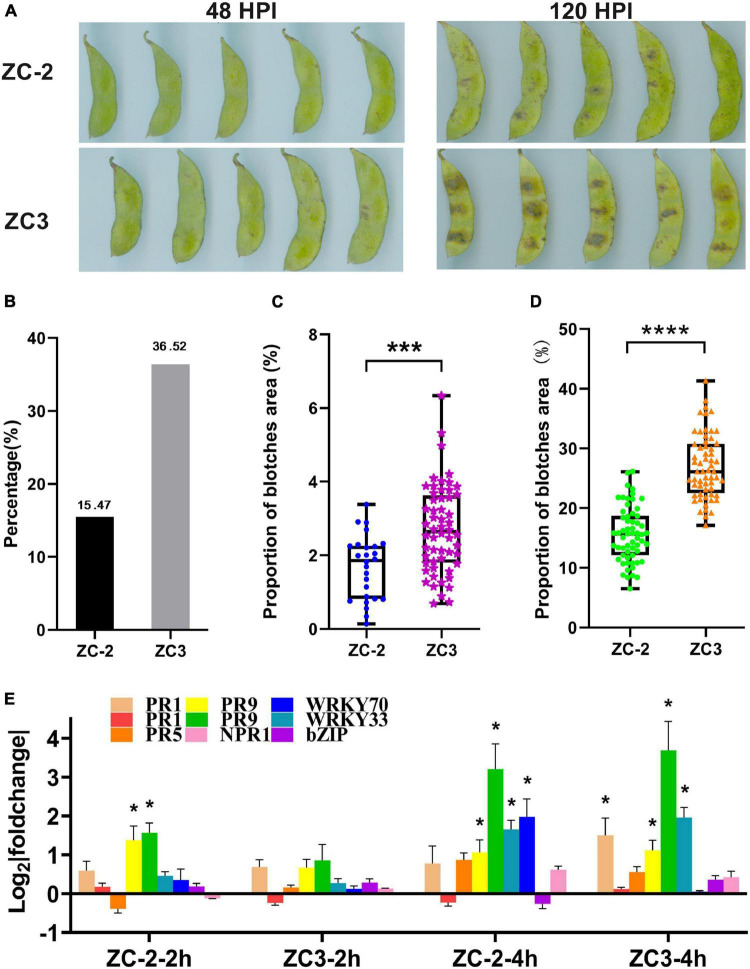
**(A)** Symptoms of anthracnose on pods of soybean lines ZC-2 and ZC3 at 2 and 5 days after inoculation with *Colletotrichum truncatum*. **(B)** Statistics on the proportion of pods with anthracnose blotches at 2 days after inoculation. **(C)** Lesion area as a percentage of total pod area for each line at 2 days after inoculation (*n* = 26, 59). ***p < 0.001. **(D)** Lesion area as a percentage of total pod area for each line at 5 days after inoculation (*n* = 60). ****p < 0.0001. **(E)** Expression levels of nine disease-responsive genes detected by quantitative real-time PCR. The *y*-axis is the log_2_ expression fold change and the *x*-axis represents the comparison groups. *p < 0.05. *PR1*, *pathogenesis-related protein 1* (*Glyma.15G062400*, *Glyma.15G062500*); *PR5*, *thaumatin-like protein* (*Glyma.10G061000*); *PR9*, *peroxidase* (*Glyma.10G222400*), (*Glyma.09G277800*); *WRKY33* (*Glyma.01G128100*); *WRKY70* (*Glyma.04G223300*); *bZIP*, *basic region-leucine zipper transcription factor* (*Glyma.05G122400*); *NPR1*, *non-expressor of pathogenesis-related genes 1* (*Glyma.15G127200*).

Symptoms of anthracnose on pods of soybean lines ZC-2 and ZC3 were recorded at 2 and 5 days after inoculation with *C. truncatum*. At 2 days after inoculation, tiny blotches had appeared on the pods of both lines (Figure 1A). However, fewer ZC-2 pods had developed blotches than ZC3 pods (Figure 1B). The proportion of the ZC-2 pod surface covered by blotches was significantly lower than that for ZC3 (Figure 1C). At 5 days after inoculation, distinct brown or black blotches had developed on the pods of ZC-2 and ZC3. The majority of lesions on the ZC-2 pods were tiny spots, whereas large lesions were common on the ZC3 pods (Figure 1A). The proportion of the ZC-2 pod surface covered by blotches was significantly lower than that for ZC3 (Figure 1D). These results indicated that the anthracnose resistance of ZC-2 was stronger than that of ZC3.

### Determination of the Sampling Time Points

The expression analysis of disease-responsive genes by qRT-PCR showed that *PR1*, *PR9*, and *WRKY* genes were significantly up-regulated at 4 HPI in ZC-2 and/or in ZC3. However, only two *PR9* genes were significantly up-regulated at 2 HPI in ZC-2 (Figure 1E). Therefore, we selected 4 HPI as the first sampling time point. At 48 HPI, tiny blotches had appeared on the pods (Figure 1A). To avoid contamination of soybean RNA-Seq data with RNAs from *C. truncatum*, due to extensive *C. truncatum* colonization of the pods, 48 HPI was selected as the last sampling time point. We included three intermediate sampling time points (8, 12, and 24 HPI).

### Transcriptome Sequencing Data Analysis

Sixty RNA libraries were sequenced. More than 2.38 billion raw reads were obtained, comprising approximately 34.19–51.85 million raw reads for each library. After performing quality control on the data, 99.59–99.72% of the reads were retained in each library. We mapped 32.83–49.98 million reads to the reference genome with a mapping rate of 95.83–96.95%, of which 93.74–95.10% were uniquely mapped and 90.35–92.36% were mapped in exon regions (Supplementary Table 2). The expression levels for 56,725 genes were calculated using the FPKM method, which resulted in identification of 33,431 expressed genes (FPKM ≥ 1), including 684 novel genes (Supplementary Table 3).

**FIGURE 2:**
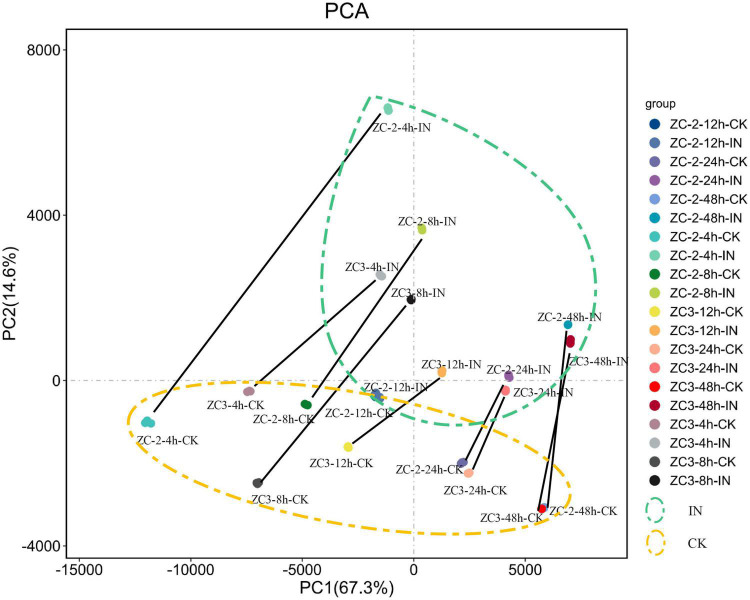
Principal component analysis scatterplot of the first and second principal components (PC1 and PC2, respectively) for gene transcripts detected from pods of soybean lines ZC-2 and ZC3 infected with *Colletotrichum truncatum* and from non-infected controls (CK) at five time points [4, 8, 12, 24, and 48 h post-inoculation (HPI)] after inoculation. Each sample comprised three biological replicates. All inoculated samples are enclosed by a green dashed ellipse and all control samples are enclosed by an orange dashed ellipse. Each control sample and its corresponding inoculated sample are connected by a black line.

The PCA showed that the three biological replicates for each sample were grouped together, which was indicative of high reproducibility, and thus that the experimental protocol and analysis methods were stable and reliable (Figure 2). All inoculated samples (enclosed by the green dashed ellipse in Figure 2) and all control samples (enclosed by the orange dashed ellipse in Figure 2) were grouped separately, and the inoculated samples were grouped to the upper right of the control samples. In addition, each inoculated sample was located to the upper right of the corresponding control sample (the corresponding samples are connected by a black line in Figure 2). These results indicated that the soybean pod transcriptome was reprogrammed in response to *C. truncatum* infection at each sampling time point, and this reprogramming showed a certain degree of consistency.

### Screening of Differentially Expressed Genes

**FIGURE 3:**
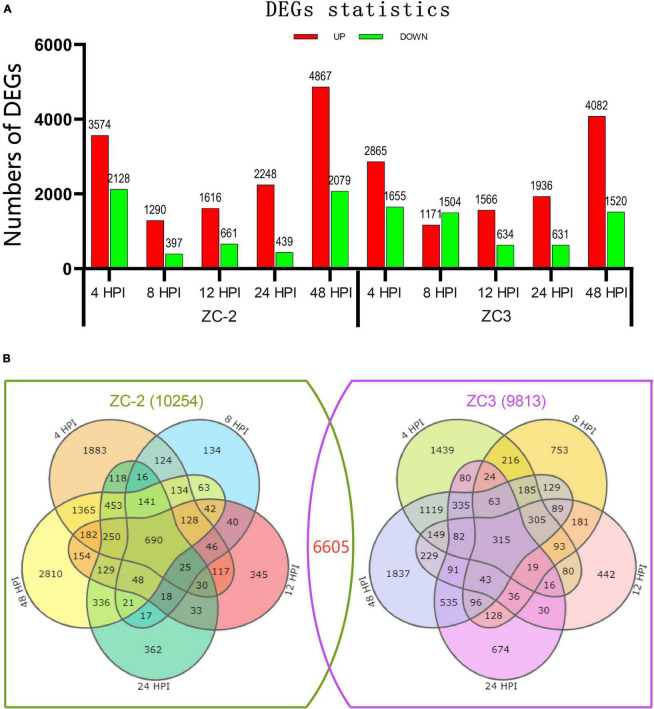
**(A)** Number of significantly differentially expressed genes (DEGs) detected from pods of soybean lines ZC-2 and ZC3 infected with *Colletotrichum truncatum*. The transcriptome was sampled at five time points after inoculation. The DEGs are shown in red (up-regulated) or green (down-regulated). **(B)** Venn diagram of the DEGs for each line.

A total of 13,462 DEGs in the ten comparison groups were screened, of which 10,254 were detected in ZC-2 and 9,813 in ZC3 (Supplementary Table 4). The DEG statistics for each comparison group are shown in Figure 3A. From 4 to 48 HPI, the number of DEGs showed a U-shaped distribution in ZC-2 and ZC (Figure 3A). That is, many more DEGs were detected at 4 and 48 HPI than at the intervening time points, which indicated that ZC-2 and ZC3 showed two large-scale response periods to *C. truncatum* infection. At 4 HPI, the pod cells had detected *C. truncatum* infection and initiated the resistance response. At 48 HPI, a fresh large-scale resistance response may have been stimulated to respond to the progression of infection with the appearance of tiny lesions on the pods at approximately 48 HPI. In addition, a higher number of DEGs were detected at these two time points for ZC-2 than for ZC3. Except for the comparison group at 8 HPI in ZC3, each comparison group contained a higher frequency of up-regulated DEGs than down-regulated DEGs, especially in the comparisons for ZC-2 (Figure 3A).

Representation of these comparative results as Venn diagrams revealed that both unique and shared DEGs occurred between and among pairs in ZC-2 and ZC3, and the DEGs of ZC-2 and ZC3 showed general correspondence (Figure 3B). Overall, 6605 genes, which accounted for 66.4 and 67.3% of the total number of DEGs in ZC-2 and ZC3, respectively, were differentially expressed in both ZC-2 and ZC3 in response to *C. truncatum* infection. For both ZC-2 and ZC3, a majority of DEGs were shared DEGs, especially those from the comparison groups at 8, 12, and 24 HPI. The similar DEGs distribution and the high proportion of shared DEGs between ZC-2 and ZC3 implied that the response patterns of ZC-2 and ZC3 to *C. truncatum* infection were highly similar on account of their highly similar genetic background.

### Validation of RNA Sequencing Expression Levels by Quantitative Real Time-PCR

**FIGURE 4:**
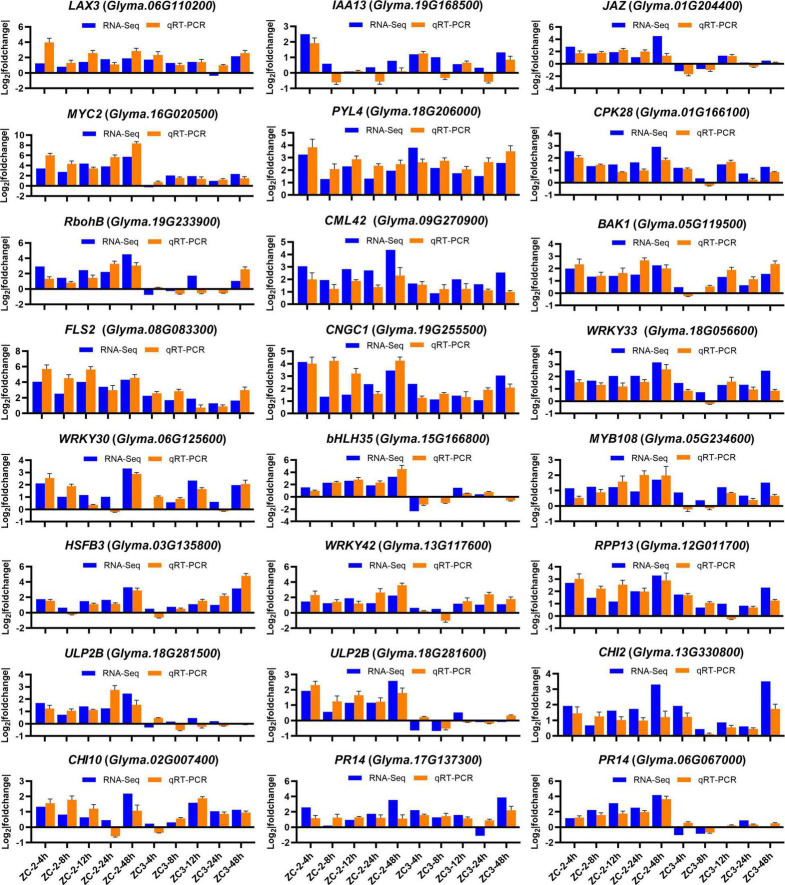
Expression levels of 24 genes detected by RNA-sequencing and quantitative real-time PCR. The *y*-axis is the log_2_ expression fold change and the *x*-axis represents the comparison groups. *LAX3*, a*uxin transporter-like protein 3*; *IAA13*, *auxin-responsive protein IAA13*; *JAZ*, *jasmonate ZIM domain-containing protein*; *PYL4*, *abscisic acid receptor PYL4*; *CPK28*, *calcium-dependent protein kinase 28*; *RobhB*, *respiratory burst oxidase protein B*; *CML42*, *calcium-binding protein 42*; *BAK1*, *BRASSINOSTEROID INSENSITIVE 1-associated receptor kinase 1*; *FLS2*, *LRR receptor-like serine/threonine-protein kinase FLS2*; CNGC1, *cyclic nucleotide-gated ion channel 1*; *HSFB3*, *heat stress transcription factor B-3*; *RPP13*, *disease resistance protein RPP13*; *ULP2B*, *disease resistance protein*; *CHI2*, *chitinase 2*; *CHI10*, chitinase 10; *PR14*, *lipid transfer-like protein*.

Twenty-four DEGs were selected to validate the RNA-Seq expression levels by qRT-PCR. The results showed a degree of reproducibility between transcript abundances assayed using RNA-Seq and the expression profiles revealed by qRT-PCR (Figure 4). However, inconsistency in abundances was suggestive of differences in sensitivity between the two methods.

### Kyoto Encyclopedia of Genes and Genomes Pathway Enrichment Analysis

**FIGURE 5:**
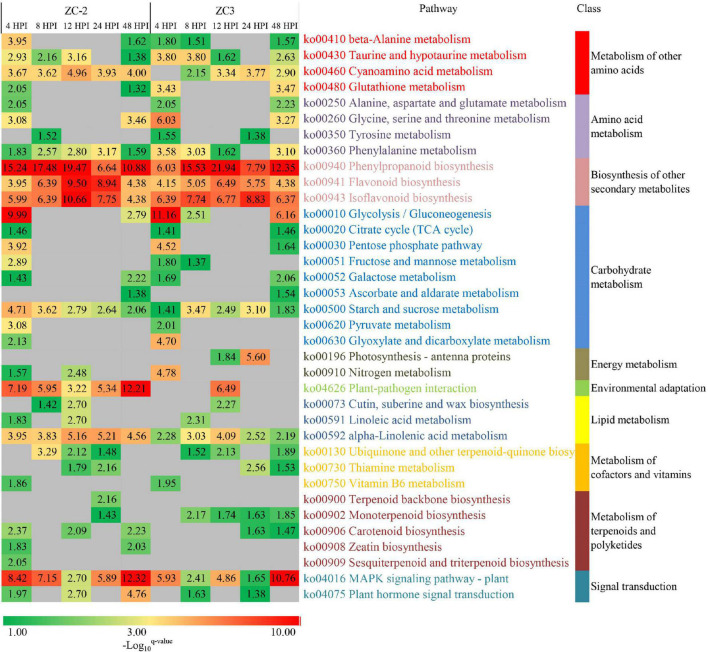
Heatmap of the enriched KEGG pathways in pods of soybean lines ZC-2 and ZC3 infected with *Colletotrichum truncatum*. The −log_10_ *q*-value is color-coded, from green to red, to indicate low to high degree of enrichment. Each row represents an enriched KEGG pathway; the columns represent each sampling time point [4, 8, 12, 24, and 48 h post-inoculation (HPI)] for ZC-2 and ZC3.

To explore the biological functions of the DEGs and the resistance mechanisms of ZC-2 and ZC3 in response to *C. truncatum* infection, KEGG pathway enrichment analysis was performed. The enriched pathways in ZC-2 and ZC3 were roughly identical, indicating that the defense response mechanisms of the two lines to *C. truncatum* infection were highly similar (Figure 5). The enriched pathways were predominantly involved in amino acid metabolism, carbohydrate metabolism, terpenoid and polyketide metabolism, phenylpropanoid biosynthesis, flavonoid biosynthesis, isoflavonoid biosynthesis, plant–pathogen interaction, and signal transduction, all of which are important components of the resistance response mechanism. In particular, the enriched pathways that showed temporal continuity, such as “cyanoamino acid metabolism,” “phenylpropanoid biosynthesis,” “flavonoid biosynthesis,” “isoflavonoid biosynthesis,” “alpha-linolenic acid metabolism,” and “starch and sucrose metabolism,” which were highly enriched in ZC-2 and ZC3, may be vital for soybean resistance to *C. truncatum* infection. The pathways specifically enriched at 4 and 8 HPI in both ZC-2 and ZC3 were largely involved in amino acid and carbohydrate metabolism. The pathways “mitogen-activated protein kinase (MAPK) signaling pathway–plant,” “plant hormone signal transduction,” “plant–pathogen interaction,” and pathways involved in metabolism of terpenoids and polyketides were responsive to *C. truncatum* infection more promptly and/or more drastically in ZC-2 than in ZC3. Therefore, these pathways may be crucial factors in the enhanced resistance of ZC-2 to anthracnose compared with that of ZC3 (Figure 5).

### Signal Transduction

#### Plant Hormone Signaling

**FIGURE 6:**
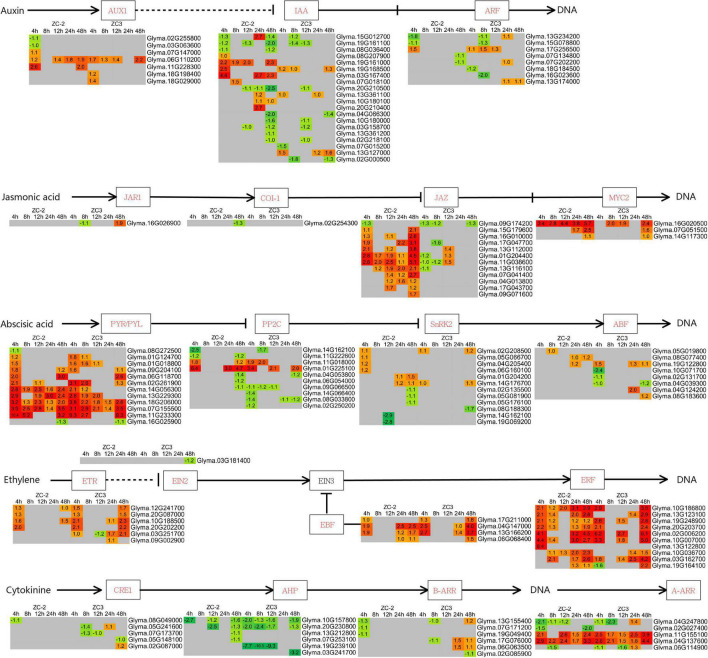
Schematic diagram of plant hormone signaling pathways together with heatmaps of the associated differentially expressed genes (DEGs). The log_2_ expression fold change is color-coded in each box: red indicates up-regulated DEGs and green indicates down-regulated DEGs. Each row represents a DEG with its gene ID; the columns represent each sampling time point [4, 8, 12, 24, and 48 h post-inoculation (HPI)] for ZC-2 and ZC3.

Genes associated with jasmonic acid (JA), auxin (AUX), abscisic acid (ABA), ethylene (ET), cytokinin (CTK), salicylic acid (SA), brassinosteroid (BR), and gibberellin (GA) signaling were differentially expressed and thus responsive to *C. truncatum* infection, although the intensity and timing of the response differed (Figure 6 and Supplementary Figure 1). The AUX, JA, ABA, ET, and CTK signaling pathways were indicated to respond more promptly and strongly to *C. truncatum* infection than the SA, BR, and GA signaling pathways. Among the former pathways, JA and AUX signaling responded to *C. truncatum* infection more rapidly and/or intensely in ZC-2 than in ZC3. Transcripts of signaling-related DEGs, such as *AUX1* (*auxin influx carrier*), *IAA* (*auxin-responsive protein IAA*), *JAZ* (*jasmonate ZIM domain-containing protein*), and *MYC2* genes, were more highly abundant in ZC-2 than in ZC3, especially at 4 and 48 HPI. The ABA, ET, and CTK signaling were strongly responsive to *C. truncatum* infection in ZC-2 and ZC3, but their response patterns differed. Almost all ET signaling-related DEGs and most ABA signaling-related DEGs were up-regulated, whereas most CTK signaling-related DEGs were down-regulated (Figure 6).

#### Mitogen-Activated Protein Kinase Signaling and Ca^2+^ Signaling

**FIGURE 7:**
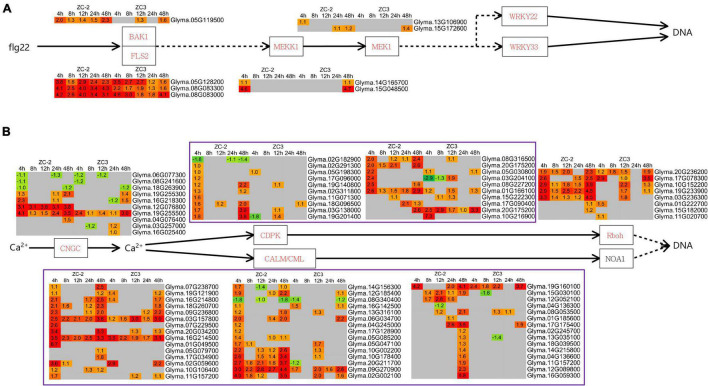
Schematic diagram of MAPK and Ca^2+^ signaling pathways together with heatmaps of the associated differentially expressed genes (DEGs). **(A)** MAPK signaling pathway; **(B)** Ca^2+^ signaling pathway. The log_2_ expression fold change is color-coded in each box: red indicates up-regulated DEGs and green indicates down-regulated DEGs. Each row represents a DEG with its gene ID; the columns represent each sampling time point [4, 8, 12, 24, and 48 h post-inoculation (HPI)] for ZC-2 and ZC3.

Integration of the “MAPK signaling pathway–plant” and “plant–pathogen interaction” pathways revealed that MAPK signaling and Ca^2+^ signaling were actively involved in the response to *C. truncatum* infection. The *MEKK1* (*mitogen-activated protein kinase kinase kinase 1*) genes responded to *C. truncatum* infection at 4 HPI in ZC-2 and at 48 HPI in ZC3, and one *MEK1* (*mitogen-activated protein kinase kinase 1*) gene responded to *C. truncatum* infection at 4 HPI specifically in ZC-2 (Figure 7A). These results indicated that MAPK signaling responded more rapidly to *C. truncatum* infection in ZC-2 than in ZC3. Ca^2+^ signaling responded positively to *C. truncatum* infection throughout the analysis period. The transcripts of a large number of Ca^2+^ signaling-related genes, including *CNGC* (*cyclic nucleotide gated channel*), *CDPK* (*calcium-dependent protein kinase*), *CALM* (*calmodulin*), and *Rboh* (*respiratory burst oxidase*) genes, were differentially expressed at each time point and the majority were up-regulated. Furthermore, transcripts of *CDPK* and *CALM* genes were more highly abundant in ZC-2 than in ZC3, especially at 4 and 48 HPI (Figure 7B). These results indicated that Ca^2+^ signaling was more sensitive to infection by *C. truncatum* in ZC-2 than in ZC3. Thus, MAPK signaling and Ca^2+^ signaling may be crucial factors for the enhanced resistance to anthracnose of ZC-2 compared with that of ZC3.

### Differentially Expressed Transcription Factors, Putative Resistance Genes, and Pathogenesis-Related Genes

**FIGURE 8:**
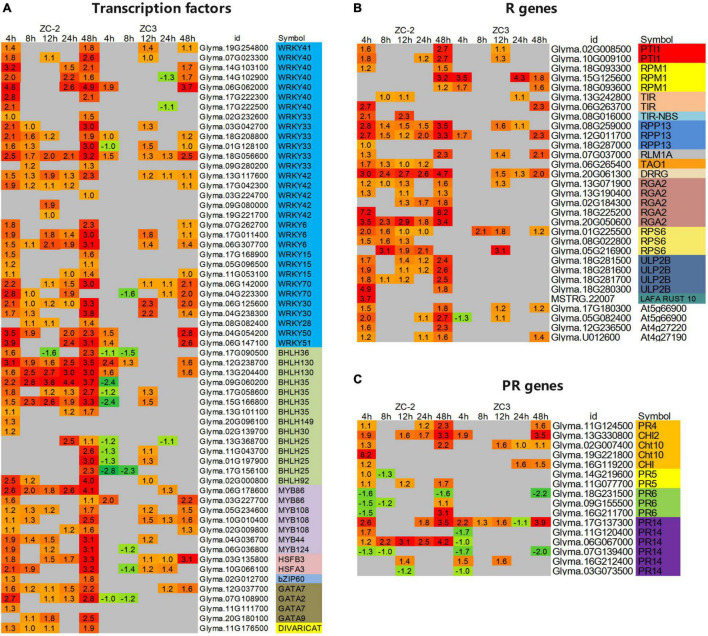
Heatmaps of differentially expressed **(A)** transcription factors, **(B)** resistance (R) genes, and **(C)** pathogenesis-related (PR) genes that showed a differential response to infection with *C. truncatum* between ZC-2 and ZC3. The log_2_ expression fold change is color-coded in each box: red indicates up-regulated differentially expressed genes (DEGs) and green indicates down-regulated DEGs. Each row represents a DEG with its gene ID; the vertical columns represent each sampling time point [4, 8, 12, 24, and 48 h post-inoculation (HPI)] for ZC-2 and ZC3.

In addition to the transcription factors involved in signal transduction, we analyzed other important differentially expressed transcription factors. Among these genes, *WRKY*, *bHLH*, and *MYB* genes were the predominant transcription factors responsive to *C. truncatum* infection. However, the response patterns of these three families of transcription factors to *C. truncatum* infection were not identical. For example, almost all *WRKY* genes were up-regulated, whereas almost one-third of the *bHLH* genes and half of the *MYB* genes were up-regulated (Figure 8A and Supplementary Figure 2).

In addition, certain transcription factors responded differentially to infection by *C. truncatum* between ZC-2 and ZC3. For instance, many *WRKY* and *MYB* transcription factors responded to *C. truncatum* infection specifically in ZC-2, especially at 4 and 8 HPI, including *WRKY6*, *WRKY15*, *WRKY33*, *WRKY40*, *WRKY42*, *MYB86*, and *MYB108*. The majority of differentially expressed *bHLH* genes were identified in both ZC-2 and ZC3. Most were strongly up-regulated in ZC-2 but down-regulated in ZC3, such as *bHLH25* and *bHLH35*, or relatively weakly up-regulated in ZC3, such as *bHLH36* and *bHLH130*, especially at 4 HPI. Several other transcription factor families showed a positive response to *C. truncatum* infection, such as *HSF* (*heat stress transcription factor*), *bZIP* (*homeobox-leucine zipper protein*), and *GATA* genes (Figure 8A). In conclusion, the differential responses of the aforementioned transcription factors to *C. truncatum* infection between ZC-2 and ZC-3 may be a contributing factor in the differential resistance to anthracnose observed between the two soybean lines.

A large number of putative R and PR genes were identified in response to *C. truncatum* infection, of which most, especially putative R genes, were strongly up-regulated. Furthermore, most of these putative R and PR genes showed similar response patterns to *C. truncatum* infection (Figures 8B,C and Supplementary Figure 3). However, certain putative R genes, such as *PTI1* (*pto-interacting protein 1*), *RPP13* (*disease resistance protein RPS2*), *RGA2* (*disease resistance protein RGA2*), *RPS6* (*disease resistance protein RPS6*), *ULP2B* (*disease resistance protein ULP2B*), and PR genes, such as *CHI* (*chitinase*) and *PR14* (*lipid transfer protein*) genes, responded more promptly and strongly to *C. truncatum* infection in ZC-2 than in ZC3; this was especially the case for *ULP2B* genes, which were differentially expressed specifically in ZC-2 (Figures 8B,C). This response may be a crucial factor in the stronger resistance of ZC-2 to anthracnose than that of ZC3.

### Differentially Expressed Genes and Differentially Accumulated Metabolites Associated With Terpenoid Metabolism

**FIGURE 9:**
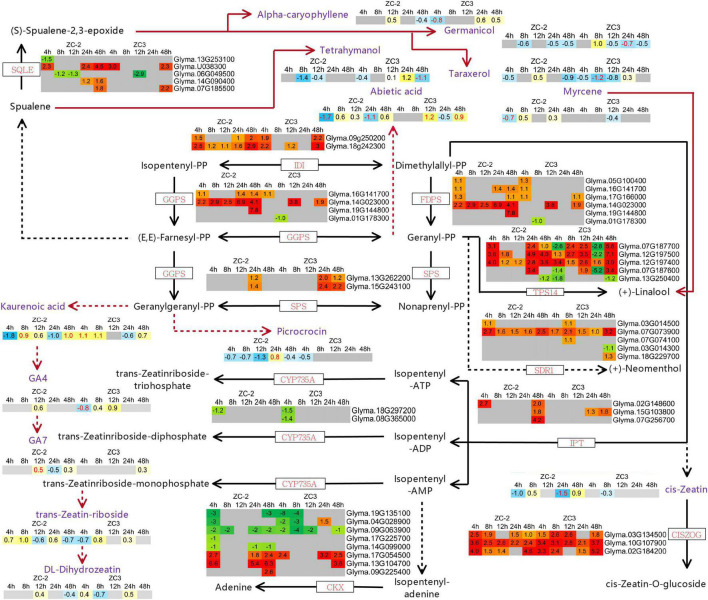
Schematic diagram of terpenoid backbone biosynthesis and zeatin biosynthesis with heatmaps of the associated differentially expressed genes (DEGs) and differentially accumulated metabolites (DAMs). The log_2_ expression fold change is color-coded in each box: red and yellow indicate up-regulated DEGs and DAMs, and green and blue indicate down-regulated DEGs and DAMs, respectively. Each row represents a DEG or DAM; the columns represent each sampling time point [4, 8, 12, 24, and 48 h post-inoculation (HPI)] for ZC-2 and ZC3. Abbreviations for DEGs are in red font and those for DAMs are in purple font.

**FIGURE 10:**
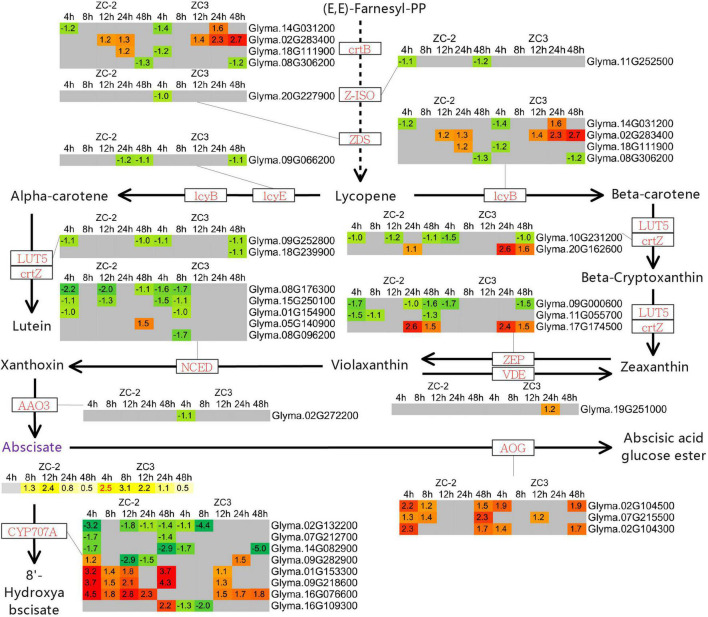
Schematic diagram of carotenoid biosynthesis with heatmaps of the associated differentially expressed genes (DEGs) and differentially accumulated metabolites (DAMs). The log_2_ expression fold change is color-coded in each box: red and yellow indicate up-regulated DEGs and DAMs, and green and blue indicate down-regulated DEGs and DAMs, respectively. Each row represents a DEG or DAM; the columns represent each sampling time point [4, 8, 12, 24, and 48 h post-inoculation (HPI)] for ZC-2 and ZC3. Abbreviations for DEGs are in red font and those for DAMs are in purple font.

The KEGG pathway enrichment analysis revealed that pathways involved in the metabolism of terpenoids and polyketides, including “terpenoid backbone biosynthesis,” “carotenoid biosynthesis,” “zeatin biosynthesis,” and “sesquiterpenoid and triterpenoid biosynthesis,” responded to *C. truncatum* infection more promptly in ZC-2 than in ZC3. Thus, we integrated these pathways and determined the associated DEGs. Most of the DEGs involved in terpenoid backbone biosynthesis and zeatin biosynthesis were up-regulated, whereas the majority of DEGs involved in carotenoid biosynthesis were down-regulated (Figures 9, 10). Most DEGs involved in the metabolism of terpenoids and polyketides showed similar response patterns to *C. truncatum* infection in ZC-2 and ZC3, whereas genes such as *GGPS* (*geranylgeranyl pyrophosphate synthase*), *FDPS* (*farnesyl diphosphate synthase*), and *TPS14* (*terpene synthase 14*) (all involved in terpenoid backbone biosynthesis), *IPT* (*adenylate isopentenyltransferase*) (involved in zeatin biosynthesis) (Figure 9), and *CYP707A* (*abscisic acid 8′-hydroxylase*) and *AOG* (*abscisate beta-glucosyltransferase*) (involved in carotenoid biosynthesis) (Figure 10), exhibited a more rapid and/or more intense and/or more prolonged response to *C. truncatum* infection. Therefore, it was speculated that timeous and intense adjustment of terpenoid backbone and plant hormone metabolism may contribute to improved soybean resistance to anthracnose.

**FIGURE 11:**
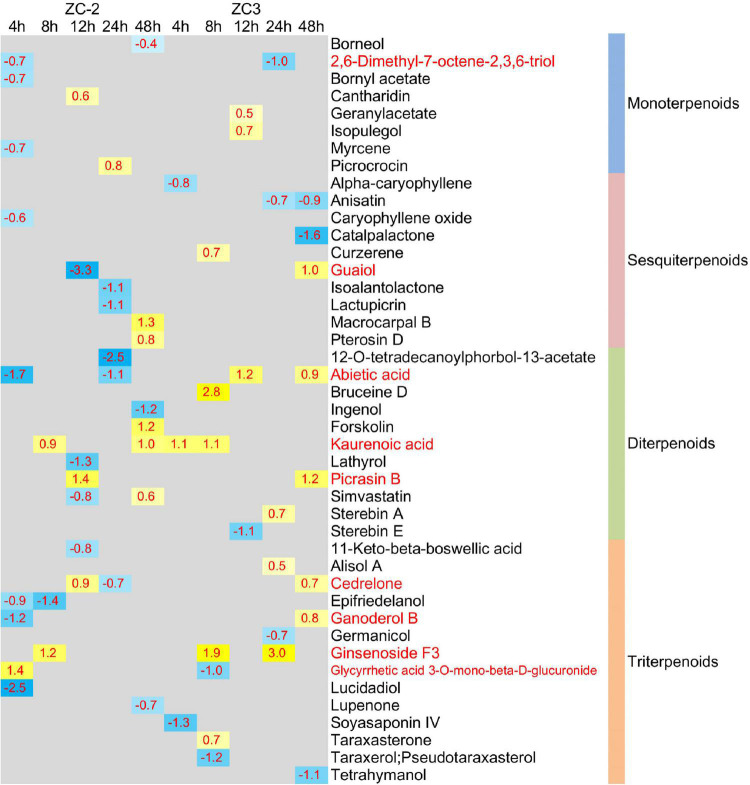
Heatmap of the significantly differentially accumulated terpenoid compounds. The log_2_ expression fold change is color-coded in each box: yellow indicates up-regulated compounds and blue indicates down-regulated compounds. Each row represents a terpenoid compound with its classification; the columns represent each sampling time point [4, 8, 12, 24, and 48 h post-inoculation (HPI)] for ZC-2 and ZC3.

In addition to plant hormones, the relative contents of 109 terpenoid compounds (4 iridoids, 23 monoterpenoids, 23 sesquiterpenoids, 24 diterpenoids, and 35 triterpenoids) were detected by UHPLC-MS (Supplementary Table 5). Forty-three terpenoid compounds (8 monoterpenoids, 10 sesquiterpenoids, 11 diterpenoids, and 14 triterpenoids) were significantly differentially accumulated (VIP > 1, *p* < 0.05) in at least one comparison group (Figure 11), of which 28 were detected in ZC-2 and 24 in ZC3. The differential accumulation of these 43 terpenoid compounds was mostly plant material-specific and time point-specific. Among these compounds, 19 and 15 compounds were differentially accumulated only in ZC-2 and ZC3, respectively, and 31 terpenoid compounds were differentially accumulated only in one comparison group. Nine terpenoid compounds were significantly differentially accumulated in both ZC-2 and ZC3 (red font in Figure 11). Except for kaurenoic acid and ginsenoside F3, the remaining seven terpenoid compounds responded to *C. truncatum* infection earlier in ZC-2 than in ZC3. Furthermore, more differentially accumulated terpenoids were identified in ZC-2 than in ZC3 at 4 HPI (Figure 11). These results demonstrated that terpenoid metabolism was highly sensitive to *C. truncatum* infection and showed timely adjustments. In addition, approximately two-thirds of the differentially accumulated terpenoid compounds in ZC-2 were down-regulated, whereas approximately two-thirds of those differentially accumulated in ZC3 were up-regulated. These results indicated that, in addition to the difference in temporal response of terpenoid metabolism between ZC-2 and ZC3, the trend for change in content also differed.

In addition to certain plant hormones (*cis*-zeatin, DL-dihydrozeatin, *trans*-zeatin-riboside, gibberellin A4, gibberellin A7, and abscisic acid), only a small number of terpenoid compounds, including myrcene, picrocrocin, alpha-caryophyllene, abietic acid, kaurenoic acid, terahymanol, taraxerol, and germanicol, were annotated in the pathways involved in terpenoid metabolism (Figures 9, 10). Genes encoding enzymes that directly catalyze the metabolism of these differentially accumulated plant hormone compounds and terpenoid compounds were not differentially expressed. The present results indicate that the transcriptomic data and UHPLC-MS data were not strongly correlated. The metabolism of these plant hormones and terpenoid compounds is closely associated with dimethylallyl-pyrophosphate (dimethylallyl-PP), geranyl-PP, (*E*,*E*)-farnesyl-PP, and geranylgeranyl-PP. The genes *GGPS*, *FDPS*, and *TPS14*, which exhibited a more rapid and/or more intense and/or more prolonged response to *C. truncatum* infection, are directly involved in the metabolism of dimethylallyl-PP, geranyl-PP, (*E*,*E*)-farnesyl-PP, and geranylgeranyl-PP. In this regard, the transcriptomic data and the UHPLC-MS data were somewhat similar.

### Plant Hormone and Terpenoid Compounds May Inhibit the Growth of *Colletotrichum truncatum* and/or Exogenously Increase the Resistance to Anthracnose

**FIGURE 12:**
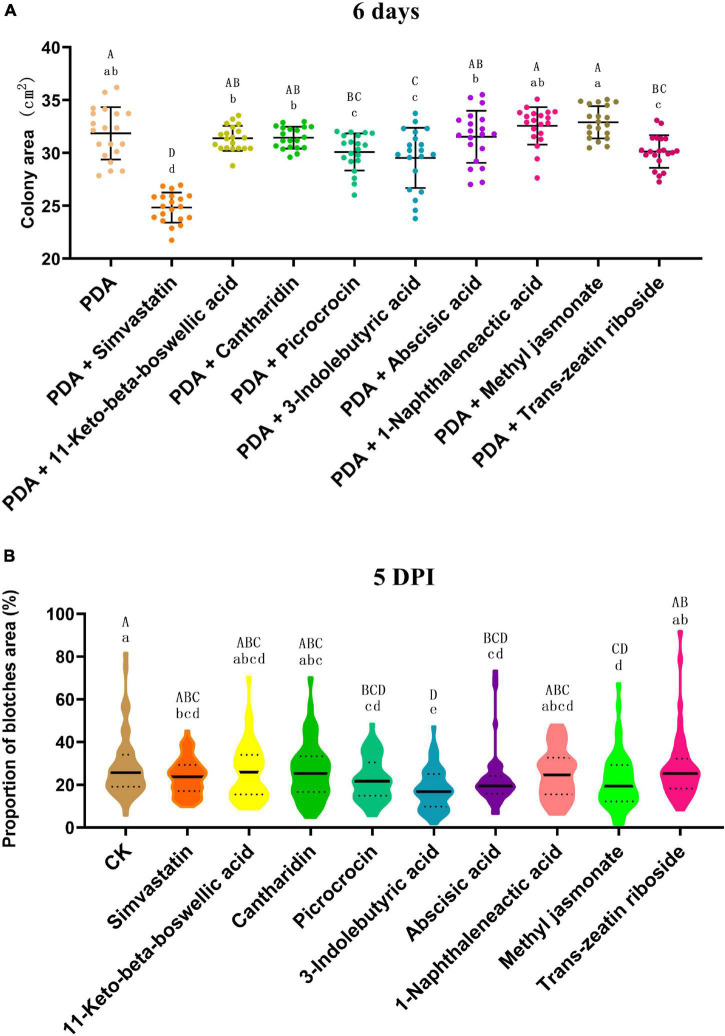
**(A)** Inhibitory effects of different terpenoids and plant hormones on the growth of *Colletotrichum truncatum*. The individual values of colony areas and the means ± SD are shown. The acolony areas were scanned and calculated with the LA-S analysis system. Different upper-case and lower-case letters indicate statistical significance at *p* < 0.05 and *p* < 0.01, respectively, between individual compounds (Tukey’s HSD test, *n* = 20). **(B)** Effects of different terpenoids and plant hormones on the resistance of soybean pods to anthracnose. Violin plots of the proportion of the total blotch area and means ± SD are shown. The blotches were scanned and areas calculated with the LA-S analysis system. Different upper-case and lower-case letters indicate statistical significance at *p* < 0.05 and *p* < 0.01, respectively, between individual compounds (Tukey’s HSD test, *n* = 50).

The *in vitro* antifungal activity test revealed that the terpenoids simvastatin, picrocrocin, and *trans*-zeatin riboside, and the plant hormone 3-indolebutyric acid significantly inhibited growth of *C. truncatum*; of these compounds, simvastatin had the strongest inhibitory effect (Figure 12A and Supplementary Figure 4). In addition, exogenous application of the plant hormones 3-indolebutyric acid, abscisic acid, and methyl jasmonate, and the terpenoids simvastatin and picrocrocin significantly increased the resistance of soybean pods to anthracnose; in particular, 3-indolebutyric acid most strongly improved resistance (Figure 12B and Supplementary Figure 5). Thus, 3-indolebutyric acid, simvastatin, and picrocrocin inhibited *C. truncatum* growth and enhanced the anthracnose resistance of soybean pods.

## Discussion

The present research, involving RNA-Seq and UHPLC-MS analysis, is the first study the interaction between soybean and *C. truncatum* at the transcriptional and metabolic levels, and the molecular mechanisms of soybean resistance to anthracnose. Importantly, the results revealed several factors that contribute to the stronger resistance of ZC-2 to anthracnose than that of ZC3, including signal transduction (JA, AUX, MAPK, and Ca^2+^ signaling), transcription factors (*WRKY* and *bHLH* genes), R and PR genes (*RGA2*, *RPS6*, *ULP2B*, *CHI*, and *PR14* genes), and terpenoid metabolism, which responded more rapidly and more intensely to *C. truncatum* infection in ZC-2 than in ZC3.

The present study showed that exogenous application of 3-indolebutyric acid and methyl jasmonate, which belong to the AUX and JA classes of plant hormones, significantly increase the resistance of soybean pods to anthracnose. In addition, 3-indolebutyric acid had a significant inhibitory effect on the growth of *C. truncatum.* Therefore, JA and AUX signaling were indicated to play important roles in soybean resistance to anthracnose. Jasmonic acid, AUX, MAPK, and Ca^2+^ signaling have long been recognized as regulators of plant defense responses (Bari and Jones, 2009; Pitzschke et al., 2009; Aldon et al., 2018; Fan et al., 2020). Furthermore, there is extensive crosstalk between these signaling pathways. For example, JA and AUX signaling (Kazan and Manners, 2009; Naseem et al., 2015), JA and MAPK signaling (Liu et al., 2011), as well as JA and Ca^2+^ signaling (Lv et al., 2019), synergistically interact to mediate plant defense responses. In addition, Ca^2+^ functions in concert with other important secondary messengers, such as reactive oxygen species, which can activate the MAPK cascade and specific stress-related genes in *Arabidopsis* (Verma et al., 2016). In addition, calcium-dependent protein kinases (CDPKs) may act synergistically with the MAPK cascade in regulating pathogen-associated molecular pattern-triggered immunity signaling (Wen et al., 2020). Therefore, we hypothesize that coordination of these signaling pathways in response to *C. truncatum* infection may contribute to the improved resistance of ZC-2 to anthracnose.

The transcription factors *WRKY6*, *WRKY15*, *WRKY33*, *WRKY40*, *WRKY41*, *WRKY42*, *WRKY70*, *bHLH13*, *bHLH25*, and *bHLH35* responded more rapidly and more intensely to *C. truncatum* infection in ZC-2 than in ZC3. Plant WRKY transcription factors are crucial regulatory components of plant responses to pathogen infection (Jiang et al., 2017). WRKY transcription factors might modulate and perceive upstream signals, and regulate the expression of resistance-associated genes and camalexin synthesis in response to pathogen infection. Thus, we were curious about the regulatory relationship between these WRKY transcription factors and JA, AUX, MAPK, and Ca^2+^ signaling. In pepper (*Capsicum annuum* L.) *CaWRKY6* and *CaWRKY40* activate the plant defense response by perceiving JA signaling (Hussain et al., 2019). *AtWRKY40* stimulates JA signaling *via* suppression of JASMONATE ZIM-DOMAIN repressors in *Arabidopsis* roots in response to *Trichoderma* colonization (Brotman et al., 2013). *Arabidopsis AtWRKY33* positively regulates the JA signaling pathway upon challenge by *Botrytis cinerea* Pers. (Birkenbihl et al., 2012). *GhWRKY70D13* negatively regulates resistance to *Verticillium dahliae* Kleb. in cotton (*Gossypium hirsutum* L.) *via* ET and JA biosynthesis and signaling pathways (Xiong et al., 2020). Therefore, it is reasonable to consider that JA signaling and the aforementioned WRKY transcription factors, both of which respond to *C. truncatum* infection rapidly and intensely in ZC-2, participate in regulatory mechanisms to improve soybean resistance to anthracnose. However, the specific mechanisms involved require further investigation.

Extensive crosstalk between Ca^2+^ signaling and WRKY transcription factors has been reported. Group IId of the WRKY protein family in plants comprises novel calmodulin (CaM)-binding transcription factors and their conserved structural motif is a Ca^2+^-dependent CaM-binding domain (Park et al., 2005). CDPKs may phosphorylate distinct substrates in the regulation of effector-triggered immunity signaling, *via* phosphorylation of WRKY transcription factors involved in immunity-related gene expression (Wen et al., 2020). It is plausible that Ca^2+^ signaling and WRKY transcription factors synergistically participate in the defense response to *C. truncatum* infection, but elucidation of the mechanism requires further research. In addition, previous research has revealed that a MAPK–WRKY pathway confers plants with resistance to pathogens (Adachi et al., 2016; Wang et al., 2018). However, information on the crosstalk between AUX signaling and WRKY transcription factors during plant–pathogen interactions is currently lacking. Zhang et al. (2008) reported that *OsWRKY31* might be a common component in the signal transduction pathways of the auxin and defense responses in rice (*Oryza sativa* L.). Crosstalk between AUX signaling and WRKY transcription factors may occur during the soybean–*C. truncatum* interaction, but this possibility needs to be explored further.

The present results indicate that R and PR genes, which were induced in response to *C. truncatum* infection, may be essential for soybean resistance to *C. truncatum* infection. Among these genes, R genes, such as *PTI1*, *RPP13*, *RGA2*, *RPS6*, and *ULP2B*, and PR genes, such as *CHI* (*chitinase*) and *PR14* (*lipid transfer proteins*) genes, may be crucial factors that enhance the resistance of ZC-2 to anthracnose compared with that of ZC3. The gene *RGA2* provides partial resistance to anthracnose in common beans (*Phaseolus vulgaris* L.) (López et al., 2003). *RPS6* confers resistance by regulating the MAPK signaling pathway in *Arabidopsis* (Takagi et al., 2019, 2020). Studies on the biological function of *ULP2B*, which was differentially expressed specifically in ZC-2, have not been reported to date.

Accumulating evidence indicates that chitinase and its enzymatic products play a critical role in plant–fungus interactions. In particular, transient expression of *CaChiIII7* increases the basal resistance to *Colletotrichum acutatum* by significantly up-regulating several defense response genes and the hypersensitive response in pepper leaves, which is accompanied by induction of hydrogen peroxide biosynthesis (Ali et al., 2020). In addition, chitinase appears to act synergistically against pathogen challenge with JA and Ca^2+^ signaling and bHLH, WRKY, and MYB transcription factors. For instance, the *Bjchitinase* gene is induced by JA but only moderately by SA (Rawat et al., 2017). Activity of the PR3b protein, a chitinase, is post-transcriptionally regulated by JA signaling (Ma et al., 2016). Plant chitinase activity may be positively or negatively regulated by Ca^2+^ (Stressmann et al., 2004; Oliveira et al., 2020). Furthermore, direct injection of Ca^2+^ triggers the expression of a chitinase gene in rice (Saito, 2003). A bHLH transcription factor is likely to be involved in the regulation of *OsChia4a* expression in a JA-dependent manner (Miyamoto et al., 2012). The transcription of *NtCHN48*, which contains two W boxes, is activated by *NtWRKY1*, *NtWRKY2*, and *NtWRKY4*, which are involved in elicitor-responsive transcription of defense genes in tobacco (Yamamoto et al., 2004). *St-WRKY1* is coregulated with a class I endochitinase during the potato–*Phytophthora infestans* (Mont.) de Bary interaction (Dellagi et al., 2000). *BjMYB1* interacts with the Wbl-4 element of *BjCHI1* to regulate plant defense against *Botrytis cinerea* (Gao et al., 2016). Similar to chitinase, lipid transfer proteins may be mutually regulated by JA and Ca^2+^ signaling (Li et al., 2008; Safi et al., 2015), as well as by WRKY and MYB transcription factors (Laquitaine et al., 2006; Cheng et al., 2020). Therefore, we confidently speculate that the timely induced expression of *chitinase* and *lipid transfer protein* genes in ZC-2 in response to *C. truncatum* infection is important to enhance resistance to anthracnose. In addition, chitinases and lipid transfer proteins may function in disease resistance through the synergistic action of JA and Ca^2+^ signaling, and bHLH, WRKY, and MYB transcription factors.

The production of defense-related metabolites is an important component of the sophisticated adaptive strategies of plants to cope with biotic stresses. The present study revealed that pathways involved in phenylpropanoid, flavonoid, isoflavonoid, amino acid, carbohydrate, and terpenoid metabolism were adjusted in response to *C. truncatum* infection (Figure 5). Undoubtedly, phenylpropanoids, flavonoids, and isoflavonoids play extremely important roles in the resistance mechanism of soybean in response to *C. truncatum* infection; the “phenylpropanoid biosynthesis,” “flavonoid biosynthesis,” and “isoflavonoid biosynthesis” pathways were highly enriched in ZC-2 and ZC3 at all time points. Furthermore, previous studies have shown that phenylpropanoids, flavonoids, and isoflavonoids contribute to increased plant resistance to anthracnose (Wang et al., 2016, 2020; Chakraborty et al., 2019; Botero et al., 2021; Jiang et al., 2021).

Compared with metabolites detected at 8, 12, and 24 HPI, the resistance responses at 4 and 48 HPI comprised an elevated number of participating metabolites, which were mainly involved in amino acid and carbohydrate metabolism (Figure 5). Amino acid and carbohydrate metabolism may provide energy for defense responses (Bolton, 2009; Kangasjarvi et al., 2012) and the substrates to produce defense-related metabolites. Certain amino acids and carbohydrates are defense-related metabolites in their own right (Zhu et al., 2018). In some cases, certain amino acids and carbohydrates function as signals to trigger defense responses (Kachroo and Robin, 2013). During the soybean–*C. truncatum* interaction, amino acid and carbohydrate metabolism were likely to participate in these roles. However, their specific roles must be clarified by more focused studies.

In the present study, transcriptomic and metabolomic data suggested that terpenoid metabolism was adjusted after *C. truncatum* infection, especially in ZC-2, which showed a rapid response to *C. truncatum* infection. The present transcriptomic data and UHPLC-MS data were not strongly correlated. We speculate that there are three reasons for this: first, there is a lag period between the metabolic and transcriptional responses to *C. truncatum* infection, but we collected samples for analysis with both methods; second, the accumulation of metabolites and the expression of the corresponding regulatory genes change to different degrees; for example, undetectable changes at the transcription level may cause measurable changes at the metabolic level; and third, changes in metabolite levels in plants in response to stress may not be completely dependent on changes in transcript levels. This study also confirmed that several terpenoids, such as simvastatin, picrocrocin, and *trans*-zeatin riboside, have significant inhibitory effects on the growth of *C. truncatum*; in addition, exogenous simvastatin and picrocrocin increased the resistance of soybean pods to anthracnose. Thus, we believe that terpenoid metabolism in response to *C. truncatum* infection may contribute to improvement in the resistance of soybean to anthracnose. Recently, a possible link between rapid induction of terpenoid metabolism and autoimmunity underlying anthracnose resistance in *Fragaria nilgerrensis* Schltdl. ex J.Gay (Mehmood et al., 2021). In addition, terpenoid metabolism may be regulated by JA signaling and bHLH, WRKY, and MYB transcription factors (He et al., 2018; Wang et al., 2021).

**FIGURE 13:**
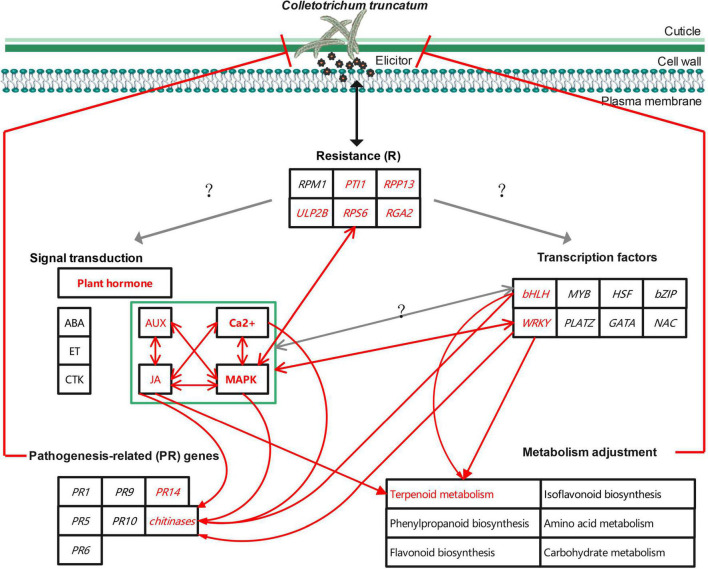
Schematic diagram of the potential mechanism to improve soybean resistance to anthracnose.

Several possible factors contributing to the stronger resistance of ZC-2 to anthracnose, compared with that of its wild-type parent ZC3, and their possible associations, have been discussed here (Figure 13), but the fundamental factors remain unknown. Previously, it was confirmed that irradiation resulted in a G→A point mutation in the genomic DNA sequence and exclusion of the entire fifth exon in the cDNA sequence of *GmIPK1* (*Glyma.14g07880*) in ZC-2 compared with ZC3, which led to reduction in the phytic acid content in ZC-2 seeds (Yuan et al., 2012). Whether the mutation of *GmIPK1* is the ultimate factor responsible for the enhanced resistance to anthracnose is unknown at present. The expression level of *GmIPK1* in soybean pods is extremely low (FPKM < 2) and *GmIPK1* is unresponsive to *C. truncatum* infection (Supplementary Table 3). Therefore, radiation mutagenesis, which is characterized by broad-spectrum variation, is likely to have induced other gene mutations in ZC-2, leading to the improvement of resistance to anthracnose. The exploitation and utilization of this key factor will be the focus of future work because it is important for elucidation of the mechanism of soybean resistance to anthracnose and to expedite the breeding of anthracnose-resistant soybean cultivars.

## Conclusion

This study revealed a potential mechanism to improve soybean resistance to anthracnose at the transcriptional and metabolic levels. Signal transduction (JA, AUX, MAPK, and Ca^2+^ signaling), transcription factors (*WRKY* and *bHLH*), resistance genes (*PTI1*, *RPP13*, *RGA2*, *RPS6*, and *ULP2B*), pathogenesis-related genes (*chitinase* and *PR14*), and terpenoid metabolism are important components of the mechanism. However, the mutual regulatory relationship between these components requires further research. *In vitro* antifungal activity and resistance induction tests confirmed that JA and AUX signaling and terpenoids, such as simvastatin, picrocrocin, and *trans*-zeatin riboside, play important roles in soybean resistance to anthracnose. In addition, the mutation of *GmIPK1* may not be the ultimate factor responsible for the enhancement of resistance to anthracnose. Thus, exploitation and utilization of mutated genes will be the focus of future work. The present research is the first attempt to explore the molecular mechanisms of soybean resistance to anthracnose, which is important for in-depth analysis of the molecular resistance mechanisms, the discovery of mutated genes or other resistance genes, and acceleration of the breeding of anthracnose-resistant soybean cultivars.

## Data Availability Statement

The datasets presented in this study can be found in online repositories. The names of the repository/repositories and accession number(s) can be found below: NCBI SRA database, accession number PRJNA800609.

## Author Contributions

FY and XF were the recipients of funding. LZ, FY, and XY conceived the experiment. LZ, QY, and HJ prepared the plant materials and collected samples. LZ, QY, HJ, and FY undertook the experiments and data analysis. LZ and FY drafted the manuscript. All authors contributed to the article and approved the submitted version.

## Conflict of Interest

The authors declare that the research was conducted in the absence of any commercial or financial relationships that could be construed as a potential conflict of interest.

## Publisher’s Note

All claims expressed in this article are solely those of the authors and do not necessarily represent those of their affiliated organizations, or those of the publisher, the editors and the reviewers. Any product that may be evaluated in this article, or claim that may be made by its manufacturer, is not guaranteed or endorsed by the publisher.

## Footnotes

1
http://www.r-project.org/
